# Ro 31‐8220 suppresses bladder cancer progression via enhancing autophagy *in vitro* and *in vivo*

**DOI:** 10.1002/2211-5463.70089

**Published:** 2025-07-21

**Authors:** Shengjun Fu, Yan Tao, Shan Wu, Yuwen Gong, Youli Zhao, Shaomin Niu, Hui Cheng, You Mu, Na Xu, Ying Wang, Jianzhong Lu, Shanhui Liu, Lanlan Li

**Affiliations:** ^1^ Insititue of Urology, Gansu Province Clinical Research Center for Urinary System Disease Lanzhou University Second Hospital Lanzhou Gansu China; ^2^ Gansu Provincial Center for Disease Control and Prevention Lanzhou Gansu China; ^3^ The Second Clinical Medical College of Lanzhou University Lanzhou University Lanzhou Gansu China; ^4^ Department of Clinical Medical Laboratory, The Second Hospital & Clinical Medical School Lanzhou University Lanzhou Gansu China; ^5^ Pediatrics Department Lanzhou University Second Hospital Lanzhou Gansu China; ^6^ Second Provincial People's Hospital of Gansu, Pathology department Affiliated hospital of Northwest Minzu University Lanzhou Gansu China

**Keywords:** apoptosis, autophagy, bladder cancer, GFP‐LC3, Ro‐31‐8220

## Abstract

Chemotherapy remains the main treatment for muscle‐invasive bladder cancer (BLCA) despite drug resistance and lack of target drugs greatly limiting long‐term survival of patients. Thus, novel and effective drugs specific to BLCA are required to aid in its treatment and improve patient survival. In the present study, we found that the compound Ro‐31‐8220, a pan‐protein kinase C inhibitor, displays potent anti‐bladder cancer efficacy *in vitro* and *in vivo*. Ro‐31‐8220 treatment suppressed bladder cancer cell migration and invasion and also induced cell apoptosis in a dose‐dependent manner. Proteomic analysis showed that Ro‐31‐8220 treatment altered the expression of numerous proteins and KEGG enrichment analysis demonstrated that multiple signal pathways are regulated by Ro‐31‐8220, including autophagy. To further validate these results, we carried out western blotting, GFP‐LC3 fusion protein and transmission electron microscopy analyses, all of which demonstrated that Ro‐31‐8220 induced bladder cancer cell autophagy. Blockade of autophagy with chloroquine, an autophagy inhibitor, attenuated Ro‐31‐8220 induced bladder cancer cell death. In a bladder cancer xenograft tumor growth mice model, we showed that intraperitoneal injection of Ro‐31‐8220 significantly decreased tumor size and tumor weight compared to the control group, suggesting an *in vivo* tumor suppression ability of Ro‐31‐8220 through activation of autophagy. These results suggest that Ro‐31‐8220 may be a novel promising candidate drug for bladder cancer therapy. Further studies, including clinical trials, are required to validate these results.

## Graphical abstract

The pan‐protein kinase C inhibitor Ro‐31‐8220 demonstrates potent anti‐bladder cancer effects both *in vitro* and *in vivo* by suppressing migration/invasion, inducing apoptosis and crucially activating autophagy, where blocking autophagy with chloroquine reduces its cell‐killing efficacy, suggesting its promise as a novel therapeutic candidate requiring further clinical validation.

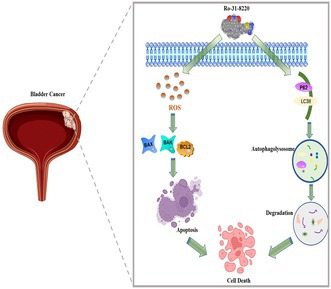

**Abbreviations**
ANOVA: analysis of variance
BLCA: bladder cancer
CCK8: cell counting kit‐8
CQ: chloroquine
CR: cisplatin resistant
KEGG: Kyoto Encyclopedia of Genes and Genomes
PBS: phosphate‐buffered saline
PKC: protein kinase C
ROS: reactive oxygen species
TEM: transmission electron microscopy

Bladder cancer (BLCA) is the 10th commonly diagnosed cancer and accounts for almost 573 000 new cases and 213 000 deaths [1]. It can be divided into non‐muscle invasive bladder cancer and muscle invasive bladder cancer according to the presence or absence of muscle invasion. Depending on the degree of metastatic cancer invasion to nearby structures, lymph nodes or distant sites, the 5‐year survival for bladder cancer patients is 77% [2]. Standards treatment for invasive or metastatic BLCA are radial cystectomy and platinum‐based adjuvant chemotherapy depending on disease type or occurrence of metastatic [3]. In addition to chemotherapy, immune checkpoint inhibitors (e.g. atezolizumab, nivolumab) and antibody‐drug conjugates (e.g. enfortumab vedotin) have been approved for metastatic bladder cancer highlighting the need for novel targeted agents [4]. However, severe challenges in bladder cancer treatment still exist as a result of its high recurrence frequency and recurrent progression. In addition, drug resistance is also frequent. Therefore, discoveries of new drugs are becoming particularly urgent.

Autophagy is a conservative lysosome‐mediated catabolic process that plays a vital role in a variety of physiological and pathological processes. It is a precisely regulated mechanism for cellular homeostasis when responding to various intracellular or excellular factors, such as starvation, hypoxia, endoplasmic reticulum stress, oxidative stress or drug treatment [5]. Autophagy is controlled in a physiological condition to maintain cellular homeostasis. However, excessive self‐degradation can be deleterious, which may lead to autophagic cell death, termed type II programmed cell death [6]. Recently, much attention has been paid on deciphering the role of autophagy in cancer and numerous studies have shown that induction or inhibition of autophagy plays a vital role in cancer progression [7, 8]. Current clinical drugs are more or less related to autophagy and targeting autophagy may have beneficial outcomes in the treatment of cancers. Therefore, there is still the need to develop new agents modulating the autophagy process for cancer therapy.

Ro‐31‐8220 is a synthetic bisindolylmaleimide derivative and pan‐protein kinase C (PKC) inhibitor that selectively targets the ATP‐binding site of PKC isoforms, demonstrating anti‐proliferative effects in preclinical cancer models. In a previous drug screening against bladder cancer cells [9], Ro‐31‐8220 exhibited a potential anti‐tumor effect; however, the underlying anti‐bladder cancer mechanisms have not been investigated. In the present study, the inhibition mechanism of Ro‐31‐8220 on bladder cancer was investigated. Using *in vitro* and *in vivo* approaches, Ro‐31‐8220 was shown to effectively inhibit bladder cancer cell viability, cell migration and colony formation. In addition, Ro‐31‐8220 induced bladder cancer cell death by enhancing excessive autophagy.

## Methods

### Chemicals and antibodies

Ro‐31‐8220 (T6643), Hoechst 33258 (T65243) and chloroquine (CQ) (T8689) were purchased from TargetMol (Boston, MA, USA). The Cell Counting Kit was purchased from Do Jindo Laboratories (Mashiki‐machi, Japan). The Annexin V‐Fluorescein Isothiocyanate Apoptosis Detection Kit (C1062L), Reactive Oxygen Species Assay Kit (S0033M), Enhanced BCA Protein Assay Kit (P0010) and Hematoxylin and Eosin Staining Kit (C0105S) were purchased from Beyotime (Shanghai, China). The primary antibodies used for immunohistochemistry and western blotting were: β‐actin (ab197345, Rabbit pAb) and BAX (ab32503, Rabbit mAb), purchased from Abcam (Cambridge, UK). LC3 (A19665, Rabbit mAb), P62 (A21702, Rabbit pAb), Beclin 1 (A21191, Rabbit mAb), ATG5 (A19677, Rabbit mAb), ATG7 (A19604, Rabbit mAb) and Ki67 (A21861, Rabbit mAb) were purchased from Abclonal (Wuhan, China). PARP (9532, Rabbit mAb), Bcl‐2 (2872, Rabbit mAb) and BAK (12105, Rabbit mAb) were purchased from Cell Signaling Technology (Danvers, MA, USA).

### Cell culture

Human bladder cancer cells T24 (SCSP‐536), UMUC‐3 (TCHu217), J82 (TCHu218) and 5637 (TCHu 1), as well as murine bladder cancer cell MB49 (YC‐C104), were obtained from the Chinese Academy of Sciences Cell Bank (Shanghai, China). Cells were cultured in RPMI‐1640 or Dulbecco's modified Eagle's medium supplemented with 10% (v:v) fetal bovine serum, 100 U·mL^−1^ penicillin and 0.1 mg·mL^−1^ streptomycin at 37 °C with 5% CO_2_.

### Cell viability assay

Cells were seeded in 96‐well plates (5 × 10^3^ cells per well) and incubated overnight before treated with different concentrations of compounds for 24 or 48 h. Then, 10 μL of cell counting kit‐8 (CCK8) reagent was added into each well and incubated for another 2 h at 37 °C. Absorbance at 450 nm was measured using a microplate reader (Thermo Fisher Scientific, Waltham, USA). Cell viability was calculated from three independent experiments. And the cell morphology was visualized using an optical microscope (Olympus, Tokyo, Japan).

### Plasmid construction and transfection

The EGFP‐LC3B plasmids containing lentivirus were produced as previously described [8]. T24 cells were infected with the EGFP‐LC3B virus and were further selected with puromycin (2 μg·mL^−1^).

### Clonogenic assay

Bladder cancer cells were grown in six‐well plates (500 cells per well) overnight to allow cells to attach. Then the cells were treated with indicated compounds for 10 days and media containing 10% fetal bovine serum and compounds were changed every 3 days. Cell colonies were fixed and stained with 0.05% (w/v) crystal violet and the number of cell colonies was counted.

### Apoptosis assays

Bladder cancer cells were treated with 0, 1.25 and 2.5 μm Ro‐31‐8220 for 24 h. Next, the cells were collected and stained with an Annexin V‐Fluorescein Isothiocyanate Apoptosis Detection Kit (Beyotime, Shanghai, China). The apoptotic and non‐apoptotic cells were separated using a Beckman CytoFLEX flow cytometer (BD Biosciences).

### Hoechst staining

Cells were plated into 12‐well plates and treated with 1.25 μm and 2.5 μm Ro‐31‐8220 for 24 h. Subsequently, the cells were fixed with 75% ethanol and stained with Hoechst 33258 working solution for 15 min in dark. After washing three times with phosphate‐buffered saline (PBS), the cells were observed and photographed with a fluorescence microscope (Olympus).

### Wound healing assay

Cells were pre‐treated with 0, 1.25, 2.5 μm Ro‐31‐8220 for 24 h. Cells were then inoculated into six‐well plates and cultured to 90% confluence. The cells were gently and slowly scratched with pipette tips and washed with PBS to remove cell debris. Cells were then further cultured with serum‐free medium. Images were captured at 0 and 24 h after scratching. The width of wound healing was quantified and compared with baseline values (time 0 h).

### 3D Matrigel drop invasion

Cells suspended in matrigel were pipetted as a droplet in 24‐well plate to allow solidification prior to adding drug‐containing solutions as previously described [10, 11]. The cells were further cultured for 6 days with media containing 10% fetal bovine serum and drugs were changed once at day 3. The area that the cell had migrated out of the matrigel drop was measured and photographed on day 6.

### Western blotting

The western blot assay was performed as previously described [8]. The original blots were cut previously before hybridization according to the protein molecular weight.

### Detection of reactive oxygen species (ROS)

Briefly, the treated cells were stained with 2′,7′‐dichlorodihydrofluorescein diacetate for 30 min in dark. After labeling, cells were washed, trypsinized and harvested in cold PBS. Cellular ROS was detected by flow cytometry [12].

### Transmission electron microscopy (TEM) analysis

The cells at logarithmic growth stage were inoculated into 60‐mm Petri dishes. After treatment with compounds for 24 h, the cells were collected, fixed, embedded and polymerized and ultrathin sections were obtained according to the relevant process [8]. Finally, the cells were visualized with a transmission electron microscopy (JEM‐1230; JEOL, Tokyo, Japan).

### Proteomics analysis

T24 cells treated with 2.5 μm Ro‐31‐8220 for 24 h were collected for TMT/iTRAQ (i.e. tandem mass tags/isobaric tags for relative and absolute quantitation) quantitative proteomics analysis. The samples were homogenized in lysis buffer and the proteins were enriched and extracted as previously described [8].

### Immunohistochemistry and histology

Paraffin‐embedded tumor tissues were examined on a 4‐μm section. After deparaffinization and repairation, the slides were stained with primary antibodies against Ki‐67 (dilution 1:500) at 4 °C overnight followed by staining with a secondary antibody. The cell nuclei were restained with hematoxylin after using diaminobenzidine. The sections were also stained with hematoxylin and eosin (H&E). and observed at 200× magnification using a microscope (Olympus).

### Tumor xenograft studies

Four‐week‐old NCG (NOD‐Prkdcem26Il2rgem26/Gpt) (GemPharmatech, Nanjing, China) female mice were kept in a pathogen‐free environment at the Experimental Animal Center in Lanzhou University Second Hospital. All animal studies and procedures have been approved and performed in accordance with the Animal Care Welfare Committee of Lanzhou University Second Hospital. Approximately 1 × 10^6^ cells were mixed with matrix gel at a ratio of 1:1. Then, a 50‐μL cell suspension was implanted subcutaneously into the dorsal flank of 0.3% pentobarbital sodium anesthetized mice. Once palpable tumors of approximately 100 mm^3^ had developed, these mice were randomized into three groups: the Ctrl (PBS), Ro‐31‐8220 (4 mg·kg^−1^) and Ro‐31‐8220 (10 mg·kg^−1^). Mice were over anesthetized (0.3%, pentobarbital sodium) and killed after the drug had been administered seven times (intraperitoneally, every 2 days), and tumor tissues were harvested for further analysis The animal study was reviewed and approved by the Ethics Committee of Lanzhou University Second Hospital (approval no. D2023‐474).

### Statistical analysis

Data were analyzed using Prism, version 6.0 (GraphPad Software Inc., San Diego, CA, USA) and are presented as the mean ± SEM. Differences between groups were analyzed by using one‐way analysis of variance (ANOVA) or an unpaired *t*‐test according to the number of groups. *P* < 0.05 was considered statistically significant. ImageJ (NIH, Bethesda, MD, USA) was used to quantify the protein expression detected using western blotting and to quantify related microscopy images.

## Results

### Ro‐31‐8220 inhibited bladder cancer cells proliferation

**Fig. 1:**
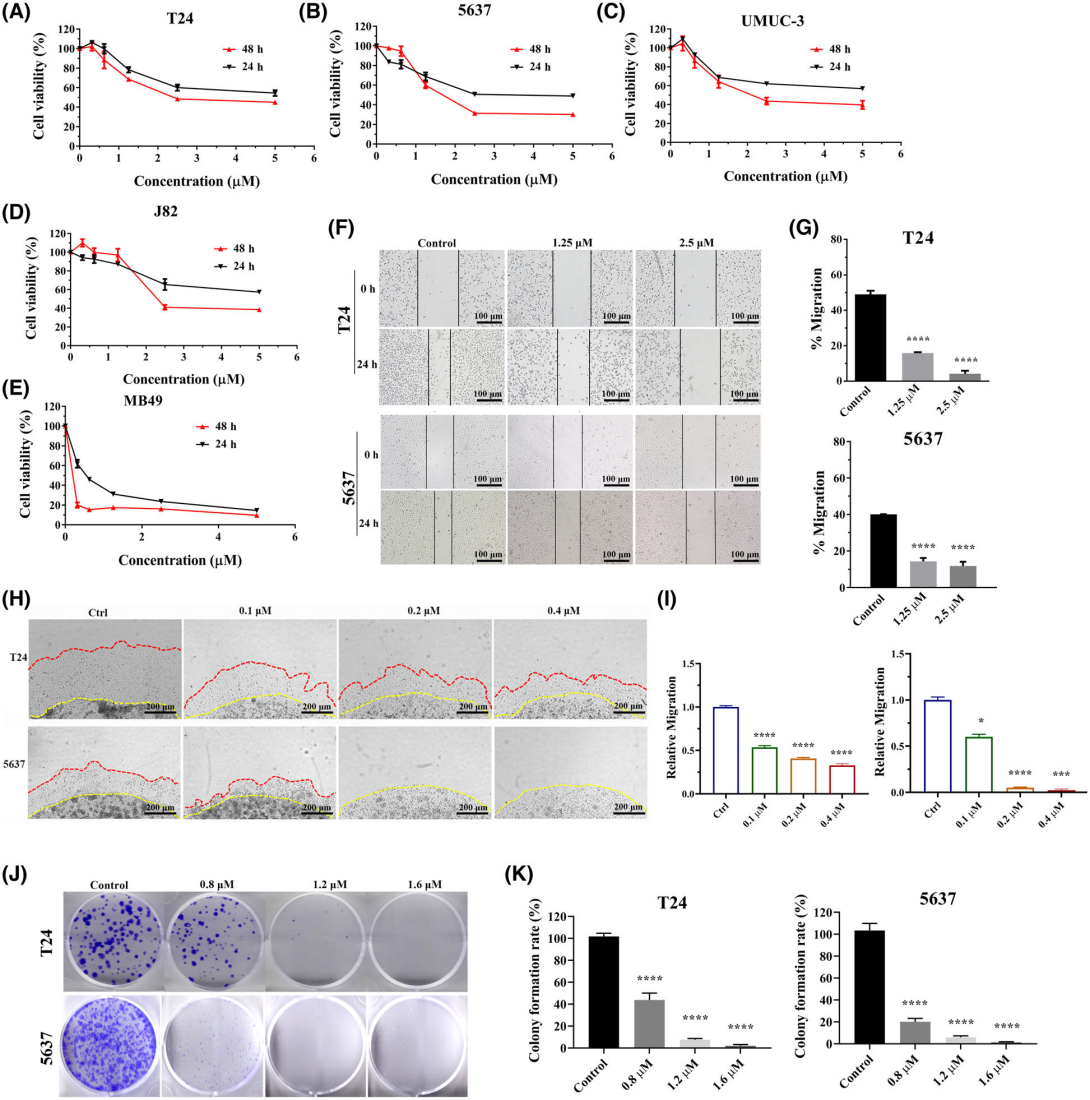
Ro‐31‐8220 inhibits bladder cancer cells. (A–E) The anti‐proliferation effect of Ro‐31‐8220 on different bladder cacner cells evaluated using the CCK8 assay (error bars indicate the SEM, *n* = 6). (F) Scratch assay (migration potential) in T24 and 5637 cells. (G) Bars represent the relative migrated distance (error bars indicate SEM, *n* = 3). (H, I) 3D Matrigel drop invasion assay (migration and invasion potential) for T24 and 5637 cells upon Ro‐31‐8220 treatment (error bars indicate the SEM, *n* = 3). (J, K) Clonogenencity assay in T24 and 5637 cells treated with Ro‐31‐8220 (error bars indicate the SEM, *n* = 3). An unpaired *t*‐test was used, *P < 0.05, **P < 0.01, ***P < 0.001, ****P < 0.0001.

To investigate the inhibitory effect of Ro‐31‐8220 on bladder cancer cells, various concentrations of the compound were administered to human bladder cancer cells (T24, 5637, J82 and UMUC‐3) or murine bladder cancers (MB49) for either 24 or 48 h. The CCK8 results indicated a significant suppression of bladder cancer cell proliferation after both 24 and 48 h compared to the control group. Furthermore, Ro‐31‐8220 demonstrated a time‐dependent and dose‐dependent inhibition of bladder cancer cell growth, with evaluated IC_50_ values below 5 μm (Fig. 1A–E and Table 1). Given the critical role of cell motility and migration in cancer progression, invasiveness and metastasis, a wound healing assay and a 3D matrigel drop invasion assay were conducted to assess the impact of Ro‐31‐8220 on bladder cancer cell migration and invasion. Treatment with Ro‐31‐8220 resulted in reduced migration of T24 and 5637 cells in the wound healing assay (Fig. 1F,G). In the 3D matrigel drop invasion assay, the distance and area the cells migrated from the matrigel drop were measured on day 6 (Fig. 1H,F,I). As expected, Ro‐31‐8220 treatment significantly inhibited bladder cancer cells migration and invasion in a dose‐dependent manner. Furthermore, a colony formation assay was conducted to confirm the inhibitory effect of Ro‐31‐8220 on bladder cancer cell proliferation, revealing a decrease in the number of colonies in a dose‐dependent manner following treatment with Ro‐31‐8220 (Fig. 1J,K). In summary, these findings indicate that Ro‐31‐8220 effectively inhibits bladder cancer cell proliferation, migration and invasion.

**Table 1:** The IC50 of Ro‐31‐8220 on bladder cancer cells.

IC_50_ (μm)	T24	5637	J82	UMUC‐3	MB49
24 h	4.71	3.76	5.92	4.09	0.57
48 h	3.13	1.89	2.98	2.56	0.11

### Ro‐31‐8220 suppressed proliferation of cisplatin‐resistant bladder cancer cells

**Fig. 2:**
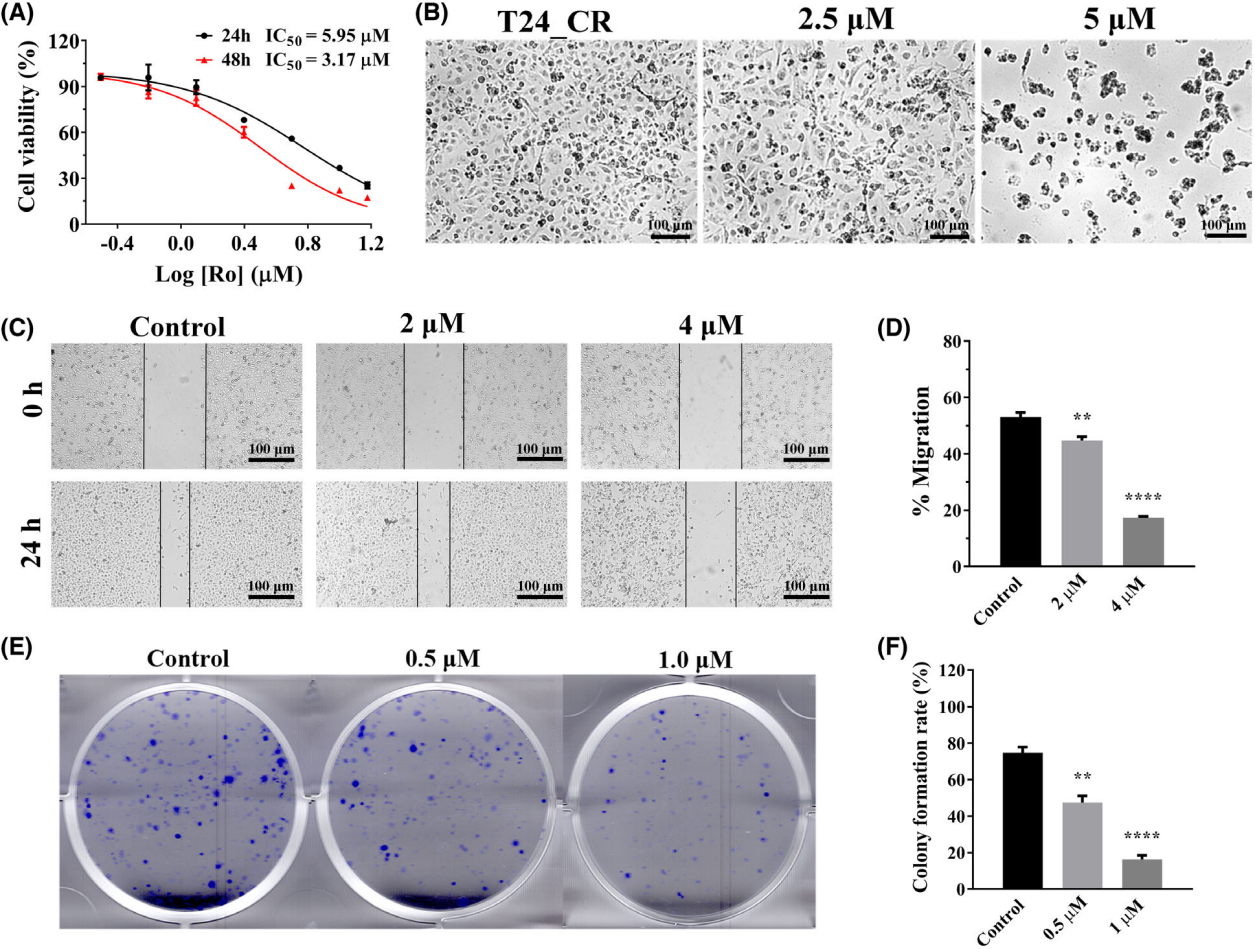
Ro‐31‐8220 restrains the growth of cisplatin resistant T24 (T24_Cis) cells. (A) Cell viability of cisplatin‐resistance T24 cells (T24_CR) after Ro‐31‐8220 treatment for 24 and 48 h (error bars indicate the SEM, *n* = 6). (B) Morphology changes of cisplatin‐resistance T24 cells after Ro‐31‐8220 treatment for 48 h. (C, D) Wound healing assay evaluated the effect of Ro‐31‐8220 on T24_CR cell migration (error bars indicate the SEM, *n* = 3). (E, F) Representative images of colony formation assay of Ro‐31‐8220 on T24_Cis cells (error bars indicate the SEM, *n* = 3). An unpaired *t*‐test was used, *P < 0.05, **P < 0.01, ***P < 0.001, ****P < 0.0001.

Cisplatin is one of the most widely used anticancer drugs in clinical bladder cancer treatment with frequently occurred drug resistance. Then cell growth inhibition ability of Ro‐31‐8220 were assessed based on our previously constructed cisplatin‐resistant T24 cells [9]. According to the CCK8 assay, Ro‐31‐8220 inhibited growth of cisplatin‐resistant T24 cells (T24_CR) in a concentration‐ and time‐dependent manner, with IC_50_ values of 5.95 μm and 3.17 μm for 24 h and 48 h, respectively (Fig. 2A). Compared with the control group, as the drug concentration increases, the cell density gradually decreases, with the cell morphology undergoing significant alterations, such as cell shrinkage, irregular shapes and cell fragmentation (Fig. 2B). Moreover, Ro‐31‐8220 could also inhibit the migration and colony formation ability of T24_CR cells assessed by a scratch assay and a colony formation assay (Fig. 2C–F). Overall, Ro‐31‐8220 has a slightly stronger killing effect on parent cells than on cisplatin‐resistant cells as indicated from the IC_50_ values.

### Ro‐31‐8220 engendered cell apoptosis in human bladder cancer cells

**Fig. 3:**
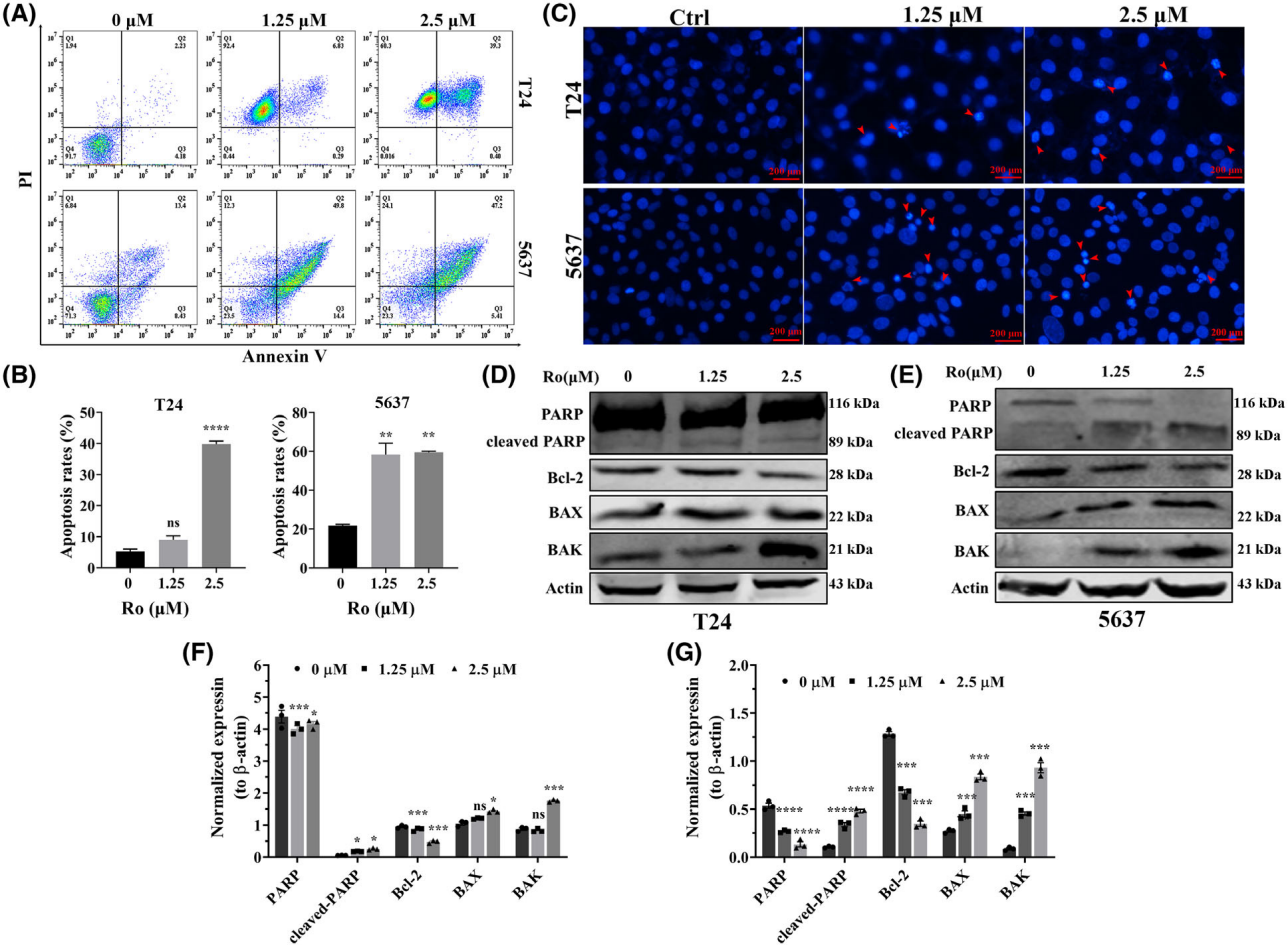
Ro‐31‐8220 induces bladder cancer cells apoptosis. Apoptosis (calculated as the sum from Q2 and Q3) in Ro‐31‐8220 treated T24 and 5637 (A, B) cells was detected using flow cytometry. (C) Hoechst 33258 dye was used to detect nuclear morphology of T24 and 5637 cells after cells were treated with or without Ro‐31‐8220 for 24 h (red arrows indicate apoptotic cells with condensed or fragmented nuclei morphology). Apoptotic protein expression in Ro‐31‐8220 treated T24 (D) and 5637 (E) cells was detected using western blotting with β‐actin as the loading control. Quantification of apoptotic protein expression of T24 (F) and 5637 (G). Data are shown as the mean ± SEM (*n* = 3) and an unpaired *t*‐test (B) and one‐way ANOVA (F, G) were used to determine significance. *P < 0.05; **P < 0.01; ***P < 0.001, ****P < 0.0001.

To thoroughly investigate the effect of Ro‐31‐8220 on the physiological function of bladder cancer cells, the apoptotic activity of bladder cancer cells after Ro‐31‐8220 treatment was determined using annexin V/propidium double staining. In comparison with the control group, Ro‐31‐8220 prominently enhanced apoptosis in all tested bladder cancer cells and the apoptosis‐inducing effect was dose dependent. Unexpectedly, Ro‐31‐8220 could induce most bladder cancer cells to the necrotic quadrant area in the flow cytometry graph, whereas inconspicuous morphology transformation was found via optical microscopy (Fig. 3A,B). Next, Ro‐31‐8220 treated T24 and 5637 cells were stained with Hoechst 33258 and nuclear morphological changes were observed by fluorescence microscopy. As can be seen, Ro‐31‐8220 treatment lead to condensed and fragmented nuclei in both T24 and 5637 cells (Fig. 3C). Subsequently, supernants of Ro‐31‐8220 treated T24 and 5637 cells were extracted and quantified for immunoblotting. As indicated in Fig. 3D,E, Ro‐31‐8220 treatment enhanced apoptosis related proteins expression, including cleaved PARP, BAX and BAK, whereas the protein expression of Bcl‐2, an anti‐apoptotic protein, was downregulated. Taken together, these results suggest that Ro‐31‐8220 enhanced bladder cancer cell death by inducing cell apoptosis.

### Ro‐31‐8220 changed cellular autophagy in bladder cancer cells

**Fig. 4:**
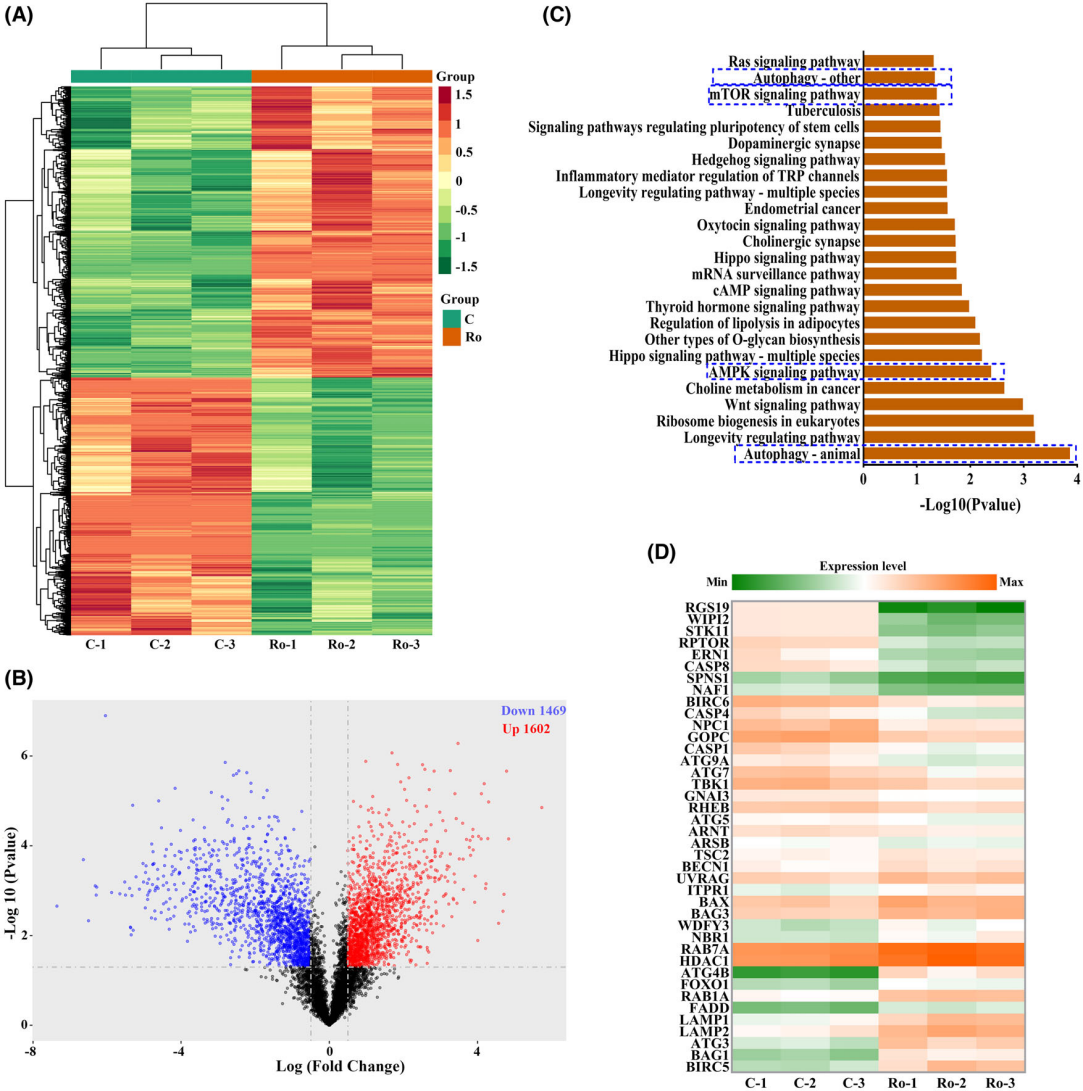
Ro‐31‐8220 treatment alters autophagy of bladder cancer cells. (A) Heat map and cluster analysis of all differentially expressed proteins in Ro‐31‐8220 treated T24 cells. (B) Volcano plots showing up‐ and down regulated proteins in the two groups. (C) Bar plot shows the top 25 KEGG signaling pathways affected by Ro‐31‐8220. Significance is was denoted as the −log_10_(*P* value). Blue dotted rectangles indicate the autophagy related signaling pathway. (D) Heatmap plot of significantly up‐regulated and down‐regulated autophagy related proteins after Ro‐31‐8220 treatment.

To depict the function of Ro‐31‐8220 in bladder cancer cells, proteomics were measured with respect to Ro‐31‐8220 pre‐treated T24 cells. As shown in Fig. 4A,B, 3089 differential expressed proteins (1602 upregulated and 1469 downregulated) were identified in Ro‐31‐8220 treated T24 cells. Furthermore, Kyoto Encyclopedia of Genes and Genomes (KEGG) (https://www.genome.jp/kegg) pathway analysis demonstrated that multiple signal pathways were regulated by Ro‐31‐8220, including autophagy, longevity regulating pathway and AMPK signaling pathway (Fig. 4C). Among them, autophagy is the most significantly enriched pathway. In addition, the highly autophagy‐related pathways, namely AMPK signaling pathway and mTOR signaling pathway, were also enriched in the KEGG analysis. This suggests that autophagy might be deeply involved in the Ro‐31‐8220 anticancer effect. Furthermore, the expression of autophagy related proteins was analyzed (Fig. 4D). Proteins involving the autophagy process were significantly regulated. Several proteins important for autophagy initiation were upregulated, such as ATG7, ATG5, ATG9 and UVRAG. In addition, proteins facilitating autophagosome maturation, autolysosome fusion, and maintaining function and stability were also dysregulated, such as BIRC6, BIR5, RAB1A, RAB7A and LAMP1/2.

### Ro‐31‐8220 treatment enhanced autophagy in bladder cancer cells

**Fig. 5:**
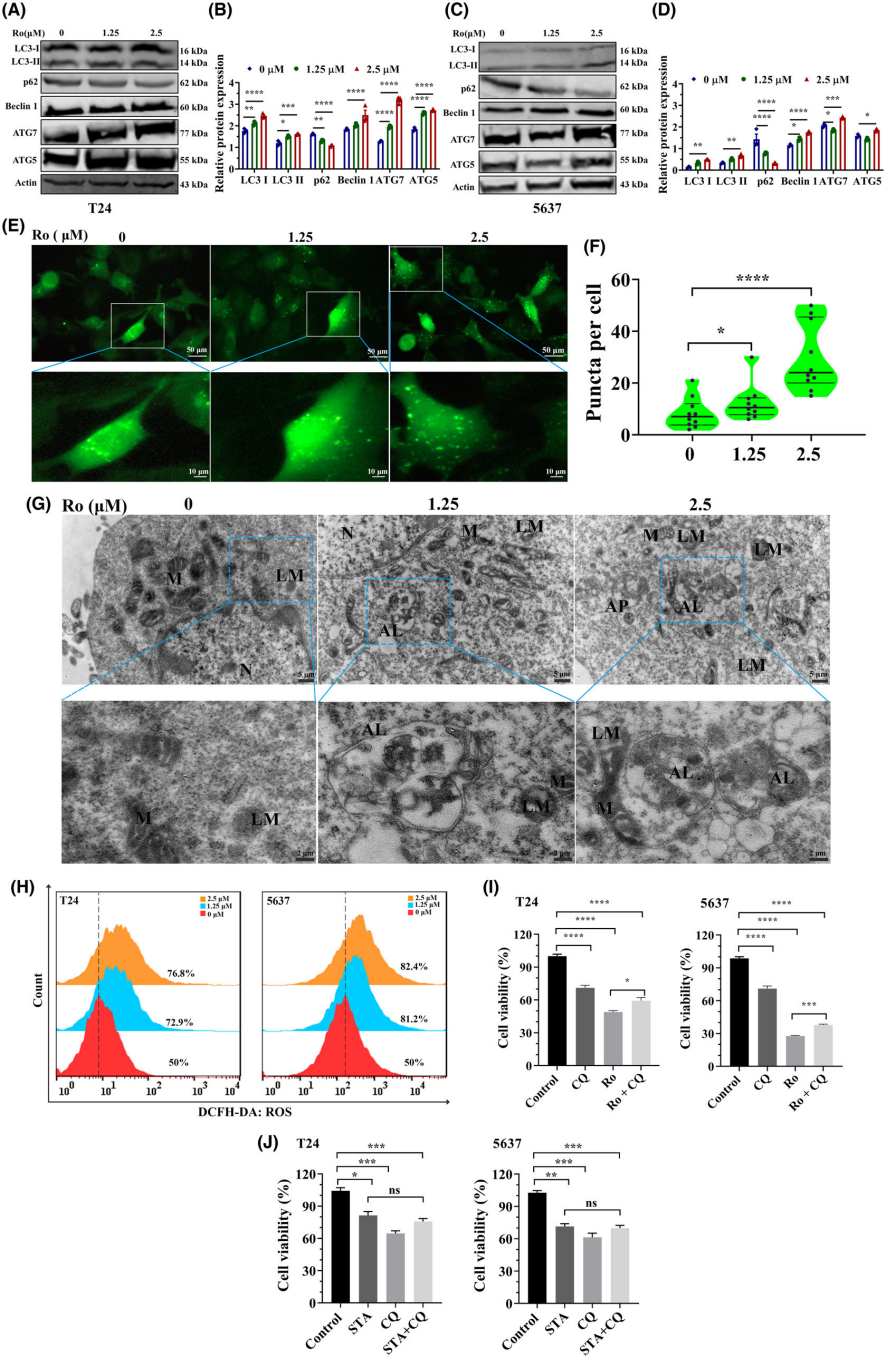
Ro‐31‐8220 induced autophagy in bladder cancer cells. (A) Protein expression of autophagy markers in T24 cells treated with Ro‐31‐8220 or not. (B) Qualification of relative protein expression in (A). (C) Protein expression of autophagy markers in 5637 cells treated with Ro‐31‐8220 or not. (D) Qualification of relative protein expression in (C). (E) Representative fluorescence images of GFP‐LC3 puncta formation in Ro‐31‐8220 treated T24 cells. (F) Quantification of GFP‐LC3 puncta per cell. (G) TEM images of T24 cells treated with Ro‐31‐8220 or not. AP, autophagosomes; AL, autolysosomes; M, mitochondria; N, nuclear; LM, Lysosome. (H) Cellular ROS level in Ro‐31‐8220 treated T24 cells and 5637 cells were measured using 2′,7′‐dichlorodihydrofluorescein diacetate dye. (I) Cell viability of T24 and 5637 cells treated with Ro‐31‐8220 (2.5 μm) with CQ added or not for 48 h was measured using the CCK8 assay. (J) Cell viability of T24 and 5637 cells treated with PKC inhibitor staurosporine (1.0 μm) with CQ (50 μm) added or not for 24 h was measured using the CCK8 assay. Data are shown as the mean ± SEM (*n* = 3) and one‐way ANOVA (B, D, F, J) and an unpaired *t*‐test (I) were used to determine significance. *P < 0.05; **P < 0.01; ***P < 0.001, ****P < 0.0001.

The function of Ro‐31‐8220 on bladder cancer cell autophagy was further evaluated based on multiple methods. To identify the effect of Ro‐31‐8220 on autophagy in bladder cancer cells, we monitored the expression of important autophagy markers. Ro‐31‐8220 induced significant increase and accumulation of LC3 I/II, Beclin 1, ATG5 and ATG7 proteins in T24 and 5637 cells (Fig. 5A–D). By contrast, Ro‐31‐8220 induced significant reduction in P62 protein levels, implying enhanced autophagic degradation of bladder cancer cells. In accordance with the changes of LC3‐II protein levels, GFP‐LC3 puncta structures were significantly induced by Ro‐31‐8220 treatment in stably expressing GFp‐LC3 T24 cells (Fig. 5E,F). Further evidence of Ro‐31‐8220 induced autophagy was observed using TEM ultrastructural analysis (Fig. 5G). Compared with untreated cells, a greater number of autophagesomes and auto‐lysosomes vesicles were clearly observed containing cytoplasm with high density plaques, which distinctly indicated increased autophagy. Autophagy is an evolutionarily conserved process in response to stress conditions such as abundant ROS production. Hence, intracellular ROS of Ro‐31‐8220 treated cells were measured. As expected, Ro‐31‐8220 treatment induced significantly elevated ROS in a dose‐dependent manner (Fig. 5H). To investigate whether autophagy mediated Ro‐31‐8220 induced cell death, cell viability were evaluated treated with R0‐31‐8220 in combination with autophagy inhibitor CQ or not. As expected, combination with CQ significantly reduced the inhibition effect of Ro‐31‐8220 on cell viability (Fig. 5I). To further determine the role that PKC played in Ro‐31‐8220 inhibited cell viability, PKC activity was inhibited with the inhibitor staurosporine in combination with autophagy inibitor CQ or not. The addition of CQ did not enhance or weaken the inhibition of cell viability induced by staurosporine, suggesting that Ro‐31‐8220 inhibited autophagy in bladder cancer cells is independent of its target PKC. Collectively, these results indicate that Ro‐31‐8220 enhanced autophagy in bladder cancer cells with excessive autophagy resulting in cell death.

### Ro‐31‐8220 inhibits bladder cancer progression of tumor bearing NCG mice

**Fig. 6:**
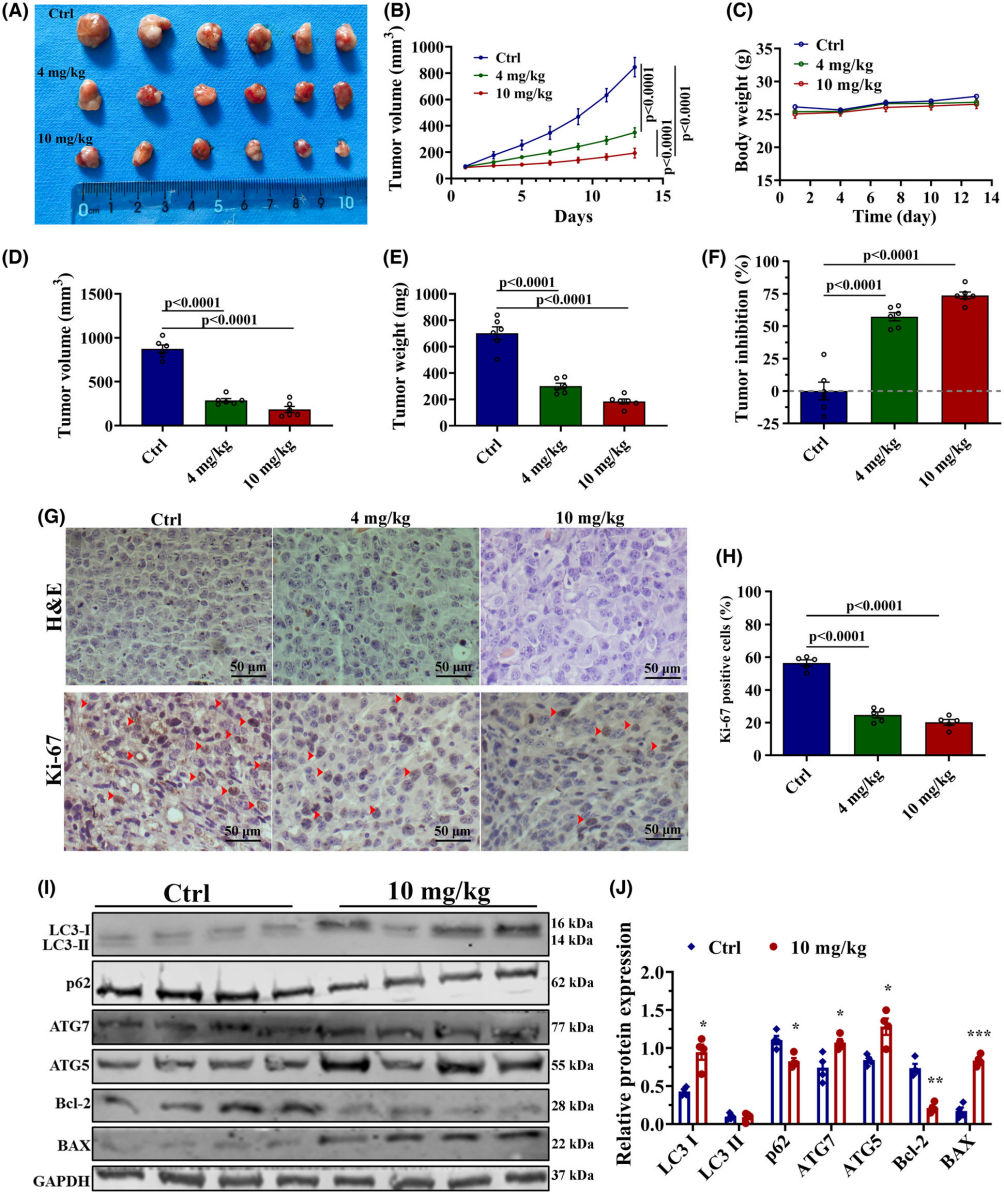
Ro‐31‐8220 arrests tumor growth of bladder cancer cells *in vivo*. Tumor images (A), tumor growth curves (B), mice body weight (C), tumor volume (D) and tumor weight (E) were recorded. (F) Inhibition rate of Ro‐31‐8220 on bladder tumor growth. (G) Tumor tissues were sectioned and subjected to hemotoxylin and eosin staining for histological morphology detection and immunohistochemical staining for Ki‐67. (H) Quantification of Ki‐67 positive cells in (G). (I) Expression of autophagy and apoptosis related proteins in mice tumor tissues. (J) Qualification of relative protein expression in (I). Data are shown as the mean ± SEM (*n* = 6) and one‐way ANOVA was used to determine significance. *P < 0.05; **P < 0.01; ***P < 0.001, ****P < 0.0001.

After confirming the efficacy of *in vitro*, we further investigated whether the treatment effect of Ro‐31‐8220 may also been detected *in vivo* model. The UMUC‐3 bladder cancer cell line was used to establish subcutaneous xenograft tumor models in the dorsal flank of NCG (NOD/ShiLtJGpt‐Prkdcem26Cd52Il2rgem26Cd22/Gpt) female mice. When tumors reached approximately 100 mm^3^, the mice were treated with PBS or Ro‐31‐8220 (4 and 10 mg·kg^−1^). Mice were killed after treatment for 15 days and the smallest size and weight of isolated tumor were found in the 10 mg·kg^−1^ Ro‐31‐8220 group (Fig. 6A–E). Both tumor size and tumor weight significantly decreased in the Ro‐31‐8220‐treated mice compared to the control group, suggesting *in vivo* tumor suppression ability of Ro‐31‐8220. No measurable side effects were observed in drug‐treated animals as revealed by body weight and lack of distressed behavior (Fig. 6C). Additionally, the Ro‐31‐8220‐treated group showed a concentration‐dependent tumor inhibition rate (Fig. 6F). Hematoxylin and eosin staining and immunohistochemical analysis of Ki‐67‐positive cells in mouse xenograft tumors confirmed that Ro‐31‐8220 administration significantly reduced the rates of cell proliferation (Fig. 6G,H). Moreover, a western blot assay confirmed the expression of autophagy markers and apoptosis proteins in mice tumor tissues (Fig. 6I,J). Together, our findings suggest that Ro‐31‐8220 can suppress bladder cancer xenograft tumor growth.

## Discussion

BLCA is one of the most common malignant tumors of the urinary system, which is a clinically heterogeneous and intractable urogenital malignant tumor with a high recurrence rate [1, 13]. In spite of novel therapies for BLCA having been developed in the past decade, cisplatin based chemotherapy is still the gold standard in perioperative and first‐line metastatic settings. However, because of chemoresistance, poor performance status, medical comorbidity and renal insufficiency, up to 50% patients are ineligible for cisplatin treatment [14, 15]. Thus, it is necessary to develop new efficient treatments for bladder cancer. Numerous studies have demonstrated that small chemicals are effective for cancer therapy as a result of their simple structure and easy synthesis [16, 17]. Targeting diverse signaling pathways with special small chemicals would offer promising benefits for cancer treatment. Previously, a small library containing 98 biological active chemicals was constructed to analyze the drug activity on bladder cancer cells [9]. According to the cell activity assay, we found that Ro‐31‐8220 could restrain the cell viability of bladder cancer cells and cisplatin‐resistant bladder cancer cells. Ro‐31‐8220 is a potent PKC inhibitor that could inhibit PKCα, PKCβI, PKCβII, PKCγ, PKCε and rat brain PKC with the IC_50_ values at the nanomolar level [18]. Although pan‐PKC inhibitors such as Ro 31‐8220 show promise, their clinical use may be associated with adverse events such as skin toxicity, gastrointestinal disturbances or cardiotoxicity, as reported in preclinical studies. In addition, several studies have demonstrated that Ro‐31‐8220 exerts an anti‐tumor effect independent of the PKC inhibition activity [19, 20]. However, there is no existing research regarding the effect of Ro‐31‐8220 on bladder cancer. In the present study, we revealed that Ro‐31‐8220 demonstrated an anti‐tumor effect in bladder cancer through inhibiting cell growth and tumor growth *in vitro* and *in vivo*. Furthermore, our results demonstrated that Ro‐31‐8220 induced excessive autophagy, which further resulted in cell death and the induction of bladder cancer cell autophagy independent of its traditional target PKC

As a vital homeostatic mechanism, the autophagy process is tightly regulated by precise coordination and interaction between various proteins. This mechanism is involved in degrading cellular contents by forming autophagosomes and fusing with lysosomes, resulting in cargo breakdown for recycling. Both excessive or insufficient autophagy will impair the autophagy process, which may hinder cell survival, including cancer cells [21, 22, 23]. LC3 is recognized as an autophagy marker with two forms, the cytosolic LC3‐I and LC3‐II. Upon autophagosomes formation, LC3‐I is lipidated and translated into LC3‐II, associated with the isolated double‐membrane phagophore [24]. SQSTM/p62, another autophagy marker, is a receptor for ubiquitinated proteins and other cargos destined for lysosomal degradation, and its level is generally inversely correlated with autophagic activity [25, 26]. Here, for the first time, we show that Ro‐31‐8220 treatment activated autophagy in bladder cancer. Upon Ro‐31‐8220 treatment, the expression of autophagic marker LC3‐II increased, whereas the expression of SQSTM/p62 decreased. Meanwhile, the expression of Beclin 1, ATG5 and ATG7 increased significantly, indicating extensive formation of autophagosomes [27, 28]. These were further substantiated by the GFP‐LC3 fusion protein plasmid and TEM with abundant GFP puncta and double‐membraned vacuoles being observed, respectively.

Numerous studies have certified that ROS regulates autophagy through modulation of various signaling pathways, such as AMPK, mTOR, Beclin‐1 and ATGs [29]. The results of most of the studies support the autophagy facilitating effect of ROS [30, 31, 32]. In the present study, we confirm that Ro‐31‐8220 significantly and dose‐dependently caused the over‐production of ROS, which resulted in autophagy activation and subsequent cell death. Furthermore, we observed an compromising cell kill activity in Ro‐31‐8220 and CQ (an autophagy inhibitor) combined treated bladder cancer cells as demonstrated by enhanced cell viability. This suggested that CQ antagonized the anti‐tumor effects of Ro‐31‐8220 in bladder cancer, indicating the antophagy‐indcuing effect of Ro‐31‐8220 on bladder cancer. In future research, we expect to identify new markers to provide guidance and assistance for the clinical treatment of bladder cancer.

## Conclusions

In summary, our study demonstrates that Ro‐31‐8220 can effectively suppress bladder cancer cell proliferation *in vitro* and *in vivo*. With various approaches, Ro‐31‐8220 was shown to play an anti‐tumor role by enhancing excessive autophagy. These results provide new insights into the mechanism for Ro‐31‐8220 mediated prohibition of bladder cancer progression.

## Conflicts of interest

The authors declare that they have no conflicts of interest.

## Author contributions

LL, SL and JL were responsible for conceptualization. SF, YT, SW, YG and YZ were responsible for methodology. HC and YM were responsible for software. YW and SN were responsible for validation. LL, JL and SF were responsible for formal analysis. YT was responsible for investigations. YZ was responsible for resources. SL, SW and NX were responsible for data curation. LL, SF and YTwere responsible for writing the original draft. SL and JL were responsible for reviewing and editing. SW and HC were responsible for visualization. LL, SL and JL were responsible for supervision. LL and SL were responsible for project administration. LL, SF, SL and JL were responsible for funding acquisition. All authors have read and approved the final version of the manuscript submitted for publication.

## Data Availability

Data for this study are available from the corresponding authors on reasonable request.
